# Nanospermidine in Combination with Nanofenretinide Induces Cell Death in Neuroblastoma Cell Lines

**DOI:** 10.3390/pharmaceutics14061215

**Published:** 2022-06-07

**Authors:** Pietro Lodeserto, Martina Rossi, Paolo Blasi, Giovanna Farruggia, Isabella Orienti

**Affiliations:** 1 Department of Pharmacy and Biotechnology, University of Bologna, Via San Donato 19/2, 40127 Bologna, Italy; pietro.lodeserto@unibo.it (P.L.); martina.rossi12@unibo.it (M.R.); p.blasi@unibo.it (P.B.); giovanna.farruggia@unibo.it (G.F.); 2 National Institute of Biostructures and Biosystems, Via delle Medaglie d’Oro 305, 00136 Rome, Italy

**Keywords:** intracellular polyamine levels, ROS increase, NLF, BR6, antitumor activity, cell motility, cell morphology, quantum phase imaging, spermidine immunomodulation

## Abstract

A new strategy to cause cell death in tumors might be the increase of intracellular polyamines at concentrations above their physiological values to trigger the production of oxidation metabolites at levels exceeding cell tolerance. To test this hypothesis, we prepared nanospermidine as a carrier for spermidine penetration into the cells, able to escape the polyamine transport system that strictly regulates intracellular polyamine levels. Nanospermidine was prepared by spermidine encapsulation in nanomicelles and was characterized by size, zeta potential, loading, dimensional stability to dilution, and stability to spermidine leakage. Antitumor activity, ROS production, and cell penetration ability were evaluated in vitro in two neuroblastoma cell lines (NLF and BR6). Nanospermidine was tested as a single agent and in combination with nanofenretinide. Free spermidine was also tested as a comparison. The results indicated that the nanomicelles successfully transported spermidine into the cells inducing cell death in a concentration range (150–200 μM) tenfold lower than that required to provide similar cytotoxicity with free spermidine (1500–2000 μM). Nanofenretinide provided a cytostatic effect in combination with the lowest nanospermidine concentrations evaluated and slightly improved nanospermidine cytotoxicity at the highest concentrations. These data suggest that nanospermidine has the potential to become a new approach in cancer treatment. At the cellular level, in fact, it exploits polyamine catabolism by means of biocompatible doses of spermidine and, in vivo settings, it can exploit the selective accumulation of nanomedicines at the tumor site. Nanofenretinide combination further improves its efficacy. Furthermore, the proven ability of spermidine to activate macrophages and lymphocytes suggests that nanospermidine could inhibit immunosuppression in the tumor environment.

## 1. Introduction

Over the last decades, polyamine research has continuously progressed, providing insight into the anabolic pathways and transport processes of polyamines. The effects of polyamines on cancer cells have also been explored in relation to different oncogenes and signaling pathways involved [1,2,3]. It has been demonstrated that oncogenes can affect the metabolism and function of polyamines by interfering with the expression and translation of key enzymes. Polyamines can also influence the expression of oncogenes in various ways, thus regulating the physiological function of cancer cells [4,5]. This research has prompted new ideas for cancer treatment. In particular, it has been demonstrated that the use of 2-difluoromethylornithine (DMFO), an inhibitor of ornithine decarboxylase (ODC), interferes with the polyamine biosynthesis and slows down the development of cancer in high-risk groups but DFMO has no significant effect on preventing cancer recurrence [6]. Moreover, the association of DFMO with inhibitors of polyamine transporters strongly improved the effect of single DFMO treatment in different tumor models by inducing significant levels of polyamine depletion in cells.

**Figure 1:**
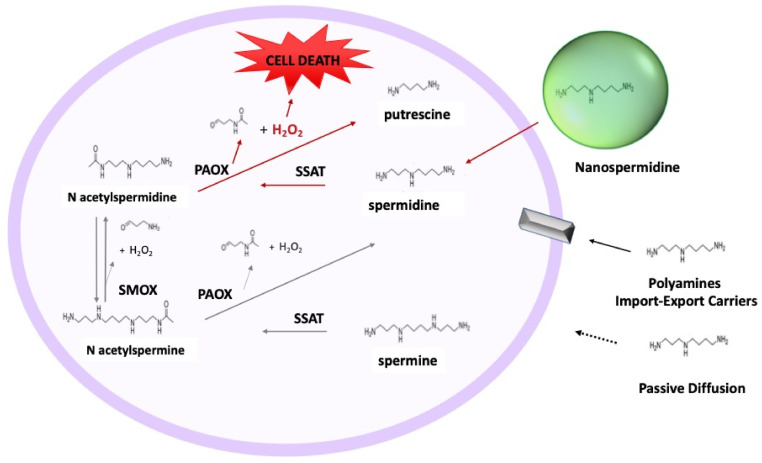
Schematic representation of the polyamine catabolic pathway and absorption mechanisms in cells. Spermidine/spermine N1-acetyltransferase (SSAT) catalyzes the acetyl-group transfer from acetyl-CoA to the aminopropyl end of spermidine or spermine, producing N1-acetylspermidine or N1-acetylspermine, respectively. These acetylated polyamines are either excreted from the cell or used as substrates for peroxisomal N1-acetylpolyamine oxidase (PAOX), producing H_2_O_2_, 3-acetoamidopropanal, and either putrescine or spermidine, depending on the starting substrate. Alternatively, N1-acetylspermine can be directly converted to spermidine by spermine oxidase (SMOX) while generating H_2_O_2_ and 3-aminopropanal. Polyamine absorption takes place by specific import-export carriers. Other possible mechanisms: passive diffusion and penetration by nanomicelles.

More recent attention has been given to polyamine analogs that upregulate polyamine catabolism and generate toxic compounds, such as H_2_O_2_, as a means to induce cancer cell death [7,8]. H_2_O_2_ is formed, with 3-acetoamidopropanal, by the acetylpolyamine oxidase (PAOX) catalyzed conversion of N-acetylspermine and N-acetylspermidine into spermidine and putrescine, respectively. It is also formed by the conversion of N-acetylspermine in N-acetylspermidine, catalyzed by the spermine oxidase (SMOX) (Figure 1). Polyamine analogs, such as N1,N11-diethylnorspermine (DENSpm), and N1-ethyl-N11-[(cyclopropyl)methyl]-4,8- diazaundecane (CPENSpm), have provided cytotoxic responses in several tumor types by their ability to upregulate SMOX and Spermidine/spermine N1-acetyltransferase (SSAT) with a consequent increase of H_2_O_2_ [9,10]. However, these molecules showed some drawbacks, such as poor specificity for cancer cells and induction of epithelial-mesenchymal transition-dedifferentiation in non-cancerous cells [11]. Overall, they showed poor positive outcomes in Phases I and II clinical trials [12].

A new strategy to cause cell death in tumors might be based on increasing intracellular polyamine concentrations above physiological values to induce the production of oxidation metabolites at levels exceeding cell tolerance (Figure 1). However, the increase in intracellular polyamine concentration cannot be easily achieved by exogenous administration because the intracellular concentration of polyamines is tightly regulated by specific transporters that import or export polyamines depending on the cell necessity [13]. In addition, passive diffusion is hampered by the low concentration gradient outside-inside the cell and the massive protonation of polyamines in body fluids [14,15,16]. For this purpose, we prepared nanospermidine by spermidine encapsulation in nanomicelles and evaluated its ability to induce cell death in two neuroblastoma cell lines: NLF and BR6. Moreover, we evaluated the combination of nanospermidine with nanofenretinide to assess if the contribution of a drug able to increase intracellular ROS levels [17,18,19,20] could improve the cytotoxic effect of nanospermidine on tumor cells. Indeed, we had previously demonstrated that nanofenretinide was highly active in neuroblastoma and DIPG tumor models, and the effects were mediated by ROS increase [21,22,23,24].

To better understand the biological effect of this treatment, we employed a new powerful microscopic technique, the quantitative phase imaging (QPI), which employs various methods (e.g., holography, ptychography) to retrieve the phase shift of light waves passing through the cells. QPI techniques measure the extent of phase delay generated by the sample and record it as pixel values within the generated image. Pixel intensity is determined by the physical thickness and the refraction index of the cells, the latter depending on biomolecule composition and organization [25,26]. In this study, a Lifecyte microscope was employed to perform QPI based on ptychography. This microscope collects multiple diffraction patterns from spatially overlapping regions of the samples to form QPI images and estimate cell number, confluence, cell dry mass, cell morphology, and motility [27,28,29,30]. Furthermore, it is built within a cell CO_2_ incubator that maintains the plate at 37 °C and 5% CO_2,_ thus allowing measures for long time periods without cell damage.

We used spermidine, among the other polyamines, as it has been proved to suppress tumorigenesis in healthy tissues and, in tumors, it contributed to the modulation of cancer-related functions, including immunoregulation, autophagy, and apoptosis [31]. In the tumor microenvironment, in particular, spermidine can induce the autophagy-dependent release of ATP, which, in turn, promotes immune surveillance [32,33]. Additionally, spermidine demonstrated the ability to alter macrophage immunometabolism and stimulate CD8+ T-cells [34] and memory B-cell responses [35,36,37].

## 2. Materials and Methods

### 2.1. Chemicals

N-4-hydroxyphenyl-retinamide (fenretinide, 4-HPR) was purchased from Olon Spa (Milan, Italy); spermidine, soy L-α-phosphatidylcholine, glyceryl tributyrate, 2-hydroxypropyl beta cyclodextrin (Mw 1460), and KOH from Sigma-Aldrich (Milan, Italy); ethanol absolute anhydrous from Carlo Erba Reagents (Milan, Italy). DMEM medium, dichlorofluorescein diacetate (DCHFDA), Hoechst 33342, glutamine, trypsine/EDTA solutions, and Human Serum were from Sigma-Aldrich.

### 2.2. Preparation of Spermidine Nanomicelles (NS) and Fenretinide Nanomicelles (NF)

Spermidine nanomicelles were prepared by mixing soy phosphatidylcholine (4 mmoles), glyceryl tributyrate (2 mmoles), spermidine (2 mmoles), 2-hydroxypropyl beta cyclodextrin (0.4 mmoles), and KOH 10 N (400 µL, 4 mmoles) to obtain a semisolid phase. Mixing to homogeneity was carried out by pressure and friction in a mortar grinder (RM 200 Retsch Verder, Italy) at 37 °C for 30 min at a 100 min^−1^ rate. The resultant semisolid phase was dispersed in PBS pH 7.4 to 50 mg/mL, filtered through 0.2 µm cellulose acetate filters (Fisher Scientific, Pittsburgh, PA, USA), and dialyzed for 72 h (dialysis membrane Mw cutoff 10 KD, Fisher Scientific) against PBS pH 7.4. The dialyzed phase was lyophilized. The dry residue was reconstituted with water and filtered again through 0.2 µm filters to obtain 50 mg/mL NS, representing the final formulative dispersion that was stored at −22 °C until use.

Fenretinide nanomicelles were prepared according to a method previously reported [21,22,23,24] by mixing soy phosphatidylcholine (4 mmoles), glyceryl tributyrate (2 mmoles), spermidine (2 mmoles), 2-hydroxypropyl beta cyclodextrin (0.4 mmoles), and KOH 10 N (400 µL, 4 mmoles) to obtain a semisolid phase. Mixing to homogeneity was carried out by pressure and friction in a mortar grinder at 37 °C for 30 min at a 100 min^−1^ rate. Fenretinide (1.2 mmoles) was dissolved in ethanol (300 µL) and KOH 10 N (120 µL, 1.2 mmole) and was subsequently added to the mixture. Homogenization was carried out in a mortar grinder for 30 min at a 100 min^−1^ rate. The resultant semisolid phase was dispersed in PBS pH 7.4 to 50 mg/mL, filtered through 0.2 µm filters, and dialyzed for 72 h (dialysis membrane Mw cutoff 10 KD) against PBS pH 7.4. The dialyzed phase was lyophilized. The dry residue was reconstituted with water and filtered again through 0.2 µm filters to obtain 50 mg/mL NF, representing the formulative dispersion that was stored at −22 °C until use.

### 2.3. Characterization of the Nanomicelles

Spermidine loading was evaluated in 50 mg/mL NS dispersions diluted (1:3, *v*/*v*) with an ethanol:water (1:1, *v*/*v*) mixture and analyzed by a fluorimetric method in comparison with the empty nanomicelles. The concentration obtained represented spermidine, both encapsulated in the nanomicelles and free in the aqueous phase. Therefore, to obtain the concentration of free spermidine, the nanomicelle dispersion was centrifuged in a 3.5 mL Ultra 5 KDa filter (Merck Millipore, Burlington, MA, USA) at 4000× *g* for 30 min and the ultrafiltrate was analyzed for spermidine content after dilution 1:3 with an ethanol:water (1:1, *v*/*v*) mixture. The difference between spermidine concentration in the nanomicelle dispersion and in the ultrafiltrate provided the concentration of the encapsulated spermidine. The nanomicelle loading was obtained as the ratio between the concentration (*w*/*v*) of the encapsulated spermidine and the concentration (*w*/*v*) of the nanomicelle dispersion.

The fluorimetric evaluation of spermidine in the nanomicelle dispersion and in the ultrafiltrate was carried out by a polyamine assay kit (Biovision, Milan, Italy) containing enzymes for the generation of hydrogen peroxide from spermidine and subsequent reaction of hydrogen peroxide with a fluorometric probe (Ex/Em = 535/587 nm) to yield a signal proportional to the amount of the polyamine present. The analysis was carried out according to the manufacturer’s instructions.

Fenretinide loading was evaluated in 50 mg/mL NF dispersions diluted (1:3 *v*/*v*) with an ethanol:water (1:1, *v*/*v*) mixture and analyzed by UV spectroscopy (Shimadzu UV-1601) at 360 nm in comparison with the empty nanomicelles. The concentration obtained represented fenretinide, both encapsulated in the nanomicelles and free in the aqueous phase. To obtain the concentration of the free drug, the nanomicelle dispersion was centrifuged in a 3.5 mL Ultra 5 KDa filter at 4000× *g* for 30 min, and the ultrafiltrate was spectrophotometrically analyzed for drug content after dilution 1:3 with an ethanol:water (1:1, *v*/*v*) mixture. The difference between the drug concentration in the nanomicelle suspension and in the ultrafiltrate provided the concentration of the encapsulated drug. The nanomicelle loading was obtained as the ratio between the concentration (*w*/*v*) of the encapsulated drug and the concentration (*w*/*v*) of the nanomicelle dispersion.

Particle size, polydispersity, and zeta potential were measured at 37 °C (Malvern Nano-ZS Spectrometer, Malvern, UK) on the nanomicelle suspensions prepared in PBS and on the nanomicelle suspensions progressively diluted up to 1:100 (*v*/*v*) starting from 50 mg/mL concentration. Dilutions were made with PBS containing 10% (*v*/*v*) Human Serum (HS) to simulate the in-vivo dilution of the nanomicelles injected into the bloodstream. A minimum of 12 measurements per sample were made. Results were the combination of 3 10-min runs for a total accumulation correlation function time of 30 min. The results were volume-weighted.

Leakage of spermidine and fenretinide from NS and NF, respectively, was measured by dialysis at 37 °C, as previously described [21,22], with some minor changes. Briefly, the nanomicelles suspensions, prepared in NaCl 0.9% (*w*/*v*) at 50 mg/mL, were diluted to 10 mg/mL with pH 7.4 PBS containing 10% HS, and 1 mL of the diluted suspension was placed in a releasing chamber separated from a receiving compartment by a dialysis membrane (Mw cutoff 5KD, Fisher Scientific). The receiving compartment was filled with 10 mL pH 7.4 PBS containing 10% HS.

Leakage from the nanomicelles was determined by evaluating spermidine or fenretinide concentrations in the receiving phase at increasing time intervals. Spermidine concentration was obtained by the polyamine assay kit previously described, and fenretinide was evaluated spectrophotometrically by its maximum absorption wavelength (360 nm). Sink conditions were monitored throughout the experiment.

### 2.4. Cell Lines

NLF and BR6 neuroblastoma cells were kindly provided by Dr. Garrett Brodeur (Children’s Hospital of Philadelphia, Philadelphia, PA, USA). These cell lines are routinely tested for integrity and authenticity, for endotoxins, mycoplasma, bacterial, and other viral contaminations, as well as genetic authenticity by multiplex PCR techniques. WS1 fibroblasts were used as a model for non-tumor cells. For the present study, the cells were grown in DMEM supplied with 10% HS, Penicillin, and Streptomycin at 37 °C in a 5% CO_2_ humidified atmosphere. They were maintained in 25 cm^2^ culture flasks (Corning, Tewksbury, MA, USA) and harvested using 0.25% Trypsin in 0.2 g/L EDTA solution.

### 2.5. MTT Assay

The tetrazolium salt 3-(4,5-dimethylthiazol-2-yl)-2,5-diphenyltetrazolium bromide (MTT) assay was used to detect cell proliferation and availability. Briefly, this assay is based on the reduction of MTT to the insoluble formazan salt by the cellular dehydrogenase. For this reason, the amount of formazan produced is considered a good indicator of the number of viable cells in the sample [38,39]. To perform the MTT assay, the cells were seeded at 10 × 10^3^ cell/cm^2^ in 96 multiwell plates, and, after 24 h, they were treated with free spermidine at concentrations varying from 10 µM to 2000 µM and with NS at nanoparticles concentrations ranging from 0.05 to 0.2 mg/mL corresponding to spermidine concentrations between 50 µM and 200 µM. Cells were also treated with fenretinide nanomicelles in combination with both free spermidine and nanospermidine. These studies were performed at constant fenretinide concentration (0.05 mg/mL nanoparticles corresponding to 10 µM fenretinide) and increasing concentrations of free spermidine (10 µM–2000 µM) or NS (nanoparticle concentrations 0.05–0.2 mg/mL corresponding to 50 µM–200 µM spermidine). After 24, 48, or 72 h, 10 µL of a 5 mg/mL MTT solution were added to each well to a final concentration of 0.5 mg/mL. After 4 h at 37 °C in the dark, 100 µL of a solution containing 10% (*w*/*v*) sodium dodecylsulfate (SDS) and HCl 0.01 mM were added to each well to dissolve the insoluble purple formazan crystals and left overnight on a shaker. The absorbance of each well was read on a TECAN plate reader (Männedorf, Switzerland) at 570 nm. To avoid the turbidity interference of biological samples, the read absorbance was normalized by a second reading at 690 nM.

### 2.6. Quantitative Phase Imaging (QPI) Microscopy

For QPI analysis, cells were seeded in a 96-well plate (Corning, Tewksbury, MA, USA) at 4 × 10^3^ per well. After 24 h, the cells were treated at the same concentrations used for the MTT assays. QPI was performed by a Livecyte microscope (Phase Focus, Sheffield, UK). Images were acquired every 60 min for 3 days using a 10× objective (0.25 NA) at 37 °C and 5% CO_2_. QPI data were analyzed using Cell Analysis Toolbox software (Phase Focus, Sheffield, UK). We evaluated cell doubling time, displacement, dry mass, sphericity to assess cell vitality, and differentiation.

### 2.7. Measurement of Intracellular ROS Level

Reactive oxygen species were detected in intact cells, according to Bergamini et al. [40]. Briefly, NLF and BR6 were seeded at the density of 1.5 × 10^4^ cells per well in a 96-well plate and incubated for 24 h to allow adhesion. Then, cells were treated for 4 h with NS at concentrations ranging from 0.05 to 0.2 mg/mL or NF at 0.05 mg/mL or with the combinations of NS and NF. Cells were then incubated with 10 µM DCFDA (Thermo Fisher Scientific, Waltham, MA, USA) for 1 h. To induce oxidative stress, cells were exposed to 150 µM tert-butyl hydroperoxide (TBH) in PBS for 30 min. Cells were then washed twice with PBS, and the fluorescence emission from each well was measured (λexc = 485 nm; λem = 535 nm) with a multi-plate reader (Enspire, Perkin Elmer, Monza, Italy). Data are reported as the mean ± SD of at least three independent experiments.

### 2.8. Confocal Laser-Scanning Fluorescence Microscopy

Confocal laser scanning microscopy (CLSM) is a type of high-resolution fluorescence microscopy that generates high-resolution images by superposition of photons emitted from the fluorescence sample and reaching the detector during one exposure period [41].

To image with CLSM, the cells were grown on glass coverslips. After 24 h, the samples were incubated at 37 °C for 30 min in the presence of 1 μg/mL Hoechst 33342 to stain cell nuclei, and, for the last 15 min, they were exposed to NS 0.2 mg/mL stained with Nile Red. The staining of NS was obtained by the addition of 1% (*w*/*w*) Nile Red to the semisolid mixture used for the preparation of NS. Reconstitution was done according to the preparation of NS nanomicelles followed by extensive dialysis to remove any residual unloaded dye. After cell exposure to Nile Red stained NS, the cells were washed with PBS 3 times, fixed with 3% formaldehyde for 10 min at room temperature, and washed repeatedly with 0.1 M glycine/PBS and PBS. As controls, the cells were exposed to Nile Red solution at the concentration corresponding to the NS treatment. Specimens were embedded in Mowiol and analyzed using a Nikon C1s confocal laser-scanning microscope, equipped with a Nikon PlanApo 40, 1.4-NA oil immersion lens. Excitation was performed at 405 nm with an argon laser and emission was recorded at 650 nm. The images were analyzed by Image J Software (version 1.53a, U. S. National Institutes of Health, Bethesda, MD, USA).

### 2.9. Statistical Analysis

All experiments were repeated at least three times on three independent samples. One-way analysis of variance (ANOVA) followed by Dunnett’s multiple comparison test was used for repeated measurement values. Differences of *p* < 0.05 were considered significant. Statistical analysis was carried out using GraphPad Prism Software (version 6.0c, GraphPad Software, San Diego, CA, USA).

## 3. Results

### 3.1. Characterization of NS and NF

**Figure 2:**
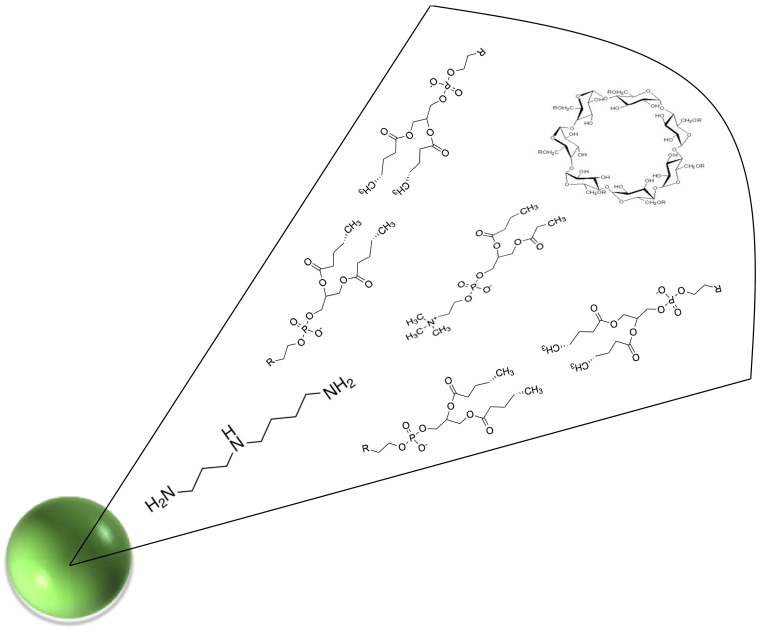
Schematic representation of the supramolecular organization of Nanospermidine main constituents: spermidine, phospholipids, 2-hydroxypropyl beta cyclodextrin.

Spermidine and fenretinide nanomicelles were obtained by dispersion in PBS of the semisolid mixtures made by phospholipids, spermidine or fenretinide, glyceryl tributyrate, and 2-hydroxypropyl beta cyclodextrin. The spontaneous self-assembling, in the aqueous phase, of the mixture components triggered nanomicelles formation and inclusion of spermidine (Figure 2) or fenretinide [21,22] in the amphiphilic nanomicelle matrix.

Particle size, polydispersity, and zeta potential were measured on the nanomicelle suspensions starting from 50 mg/mL and progressively diluting with PBS containing 10% (*v*/*v*) HS to simulate nanoparticle dilution in vivo after injection.

The mean diameter of the nanomicelles resulted optimally sized for tumor accumulation by the Enhanced Permeability and Retention (EPR) effect [42,43,44,45,46], being 148.4 ± 3.4 nm for NS and 154.1 ± 10.3 nm for NF. The polydispersity was always lower than 0.3, indicating good dimensional homogeneity (Table 1).

As is known, the EPR effect depends on the discontinuity of the tumor capillaries and the impaired lymphatic drainage in the tumor environment.

Capillary discontinuities, generally between 200 nm and 1.2 μm in diameter, have been shown to allow injected nanoparticles, smaller than 500 nm, to access tumor tissues by extravasation and accumulate due to reduced lymphatic drainage [47,48,49,50].

The surface of the nanomicelles was always negatively charged, as indicated by the zeta potential values (Table 1). Spermidine nanomicelles were characterized by the lowest absolute value of zeta potential in accordance with the alkaline character of spermidine which induces a decrease in the negative charge on the nanomicelle surface.

**Figure 3:**
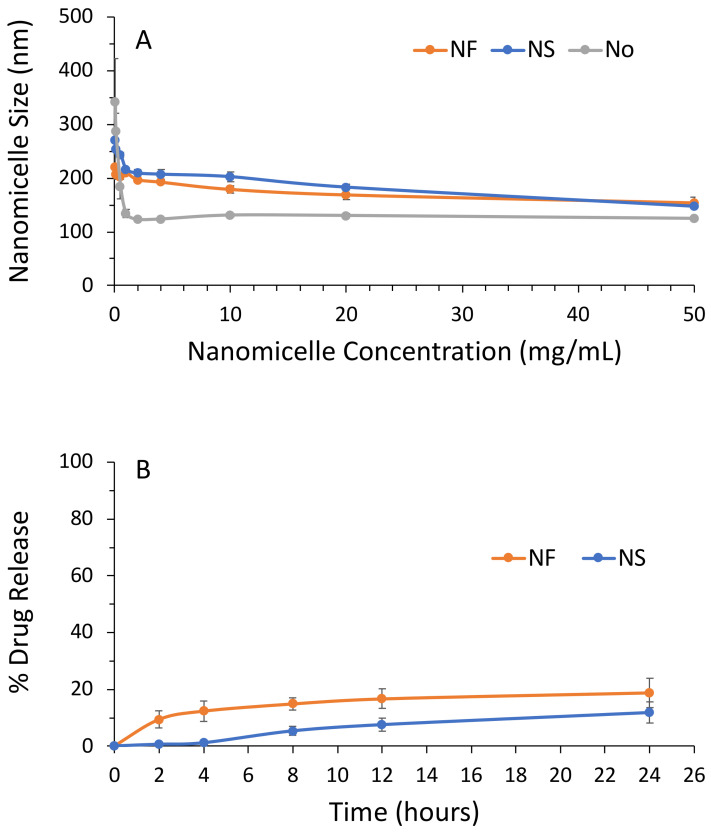
(**A**) Dimensional stability of the Nanomicelles to dilution in PBS containing 10% HS. (**B**) Stability of the Nanomicelles to leakage of their Spermidine (NS) or Fenretinide (NF) content in PBS containing 10% HS.

Dilution slightly increased the nanomicelle size (Figure 3A). The size increase was very low in the 50 to 1 mg/mL dilution range and higher between 0.5 and 0.05 mg/mL. The maximum size was obtained at 0.05 mg/mL, being 221.4 ± 23.7 nm for NF and 270.5 ± 16.1 nm for NS. This corresponded to diameter expansions of 43.67% for NF and 82.27% for NS. Size expansion at high dilutions could represent a favorable feature for nanomedicines because it could prevent retro-diffusion towards the venous circulation of the extravasated nanoparticles when their size increases due to the high dilutions provided by the tumor matrix.

Spermidine and fenretinide release from NS and NF at 24 h was 18.83% ± 5.13 and 11.82% ± 2.33, respectively (Figure 3B), indicating stability towards drug leakage in circulation for time frames longer than those required for nanoparticle accumulation in solid tumors by extravasation in accordance with the EPR effect.

### 3.2. Effect of Free Spermidine and Nanospermidine on Cell Viability

**Figure 4:**
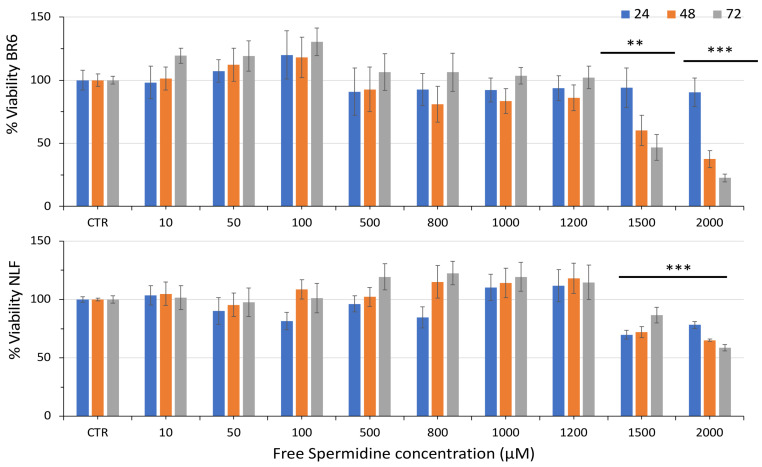
Relative viability of BR6 and NLF cells treated with increasing concentrations of Free Spermidine for 24, 48, and 72 h assessed by MTT assay. Data are presented as percentage versus control cells (100%) (mean ± SD, *n* = 6) (** *p* < 0.01, *** *p* < 0.001).

Treatment with free spermidine in the concentration range of 10 µM–2000 µM did not elicit any detrimental effects up to 1200 µM (Figure 4). The higher concentration of 1500 µM induced a slowdown of cell proliferation in NLF and a complete inhibition in BR6. At 2000 µM, free spermidine significantly reduced the 72 h cell viability in both cell lines.

**Figure 5:**
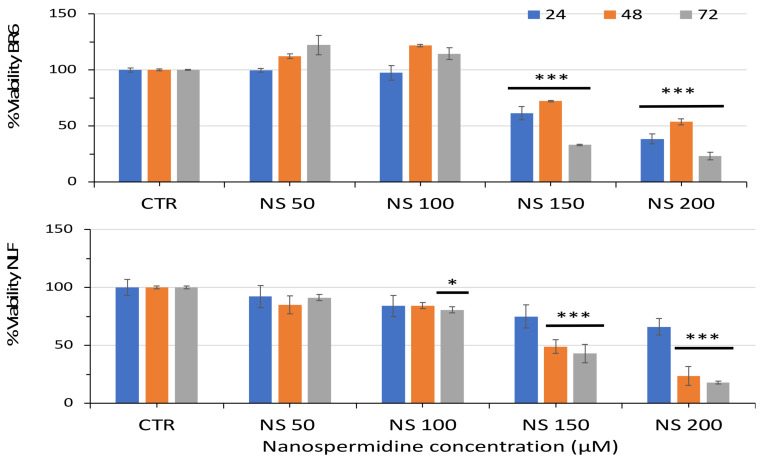
Relative viability of BR6 and NLF cells treated with increasing concentrations of Nanospermidine (NS) for 24, 48, and 72 h assessed by MTT assay. Data are presented as percentage versus control cells (100%) (mean ± SD, *n* = 6) (* *p* < 0.05, *** *p* < 0.001).

Nanospermidine had a tenfold higher effect than free spermidine in both tumor cell lines, as cell viability was significantly decreased at a nanospermidine concentration of 0.15 mg/mL corresponding to 150 µM spermidine (Figure 5).

We had previously demonstrated the antitumor activity of nanofenretinide in these cell lines [21,22]; therefore, we evaluated the combination of nanospermidine with nanofenretinide to assess if the contribution of fenretinide could improve the cytotoxic effect of nanospermidine.

**Figure 6:**
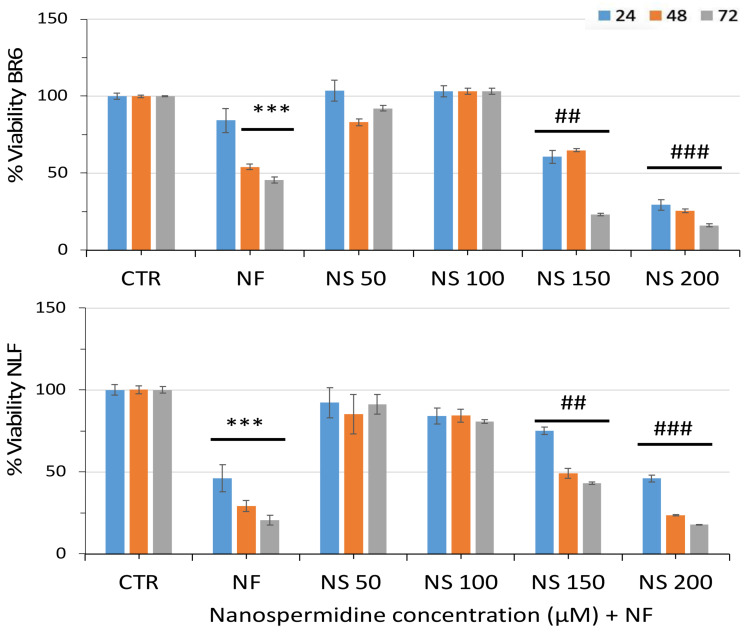
Relative viability of BR6 and NLF cells treated with increasing concentrations of Nanospermidine (NS) in the presence of Nanofenretinide (NF, 10 μM) for 24, 48, and 72 h assessed by MTT assay. Data are presented as percentage versus control cells (100%) (mean ± SD, *n* = 6) (*** *p* < 0.001) and versus NF (## *p* < 0.01, ### *p* < 0.001).

In combination with nanofenretinide, a slightly increased overall activity was obtained with nanospermidine (Figure 6) at the same concentrations that triggered cytotoxicity in single administrations: 0.15 mg/mL and 0.20 mg/mL nanospermidine, corresponding to 150 µM and 200 µM spermidine, respectively.

**Figure 7:**
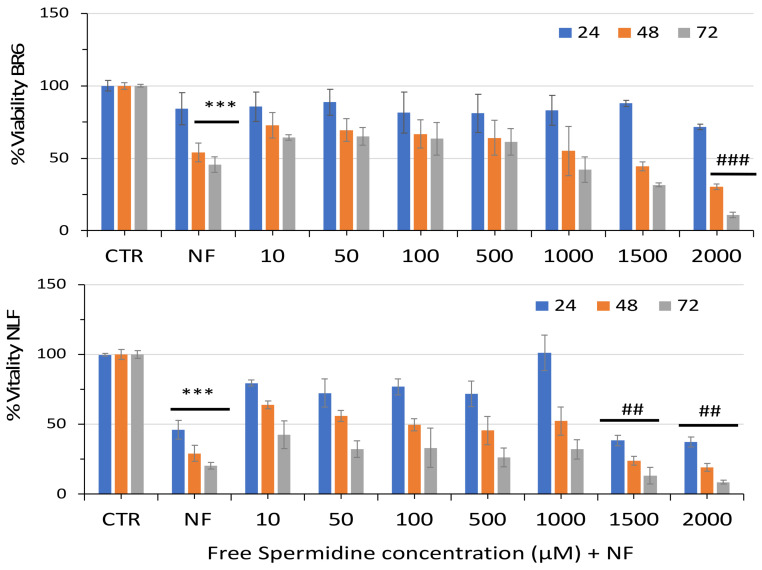
Relative viability of BR6 and NLF cells treated with increasing concentrations of Free Spermidine in the presence of Nanofenretinide (NF, 10 µM) for 24, 48, and 72 h assessed by MTT assay. Data are presented as percentage versus control cells (100%) (mean ± SD, *n* = 6) (*** *p* < 0.001) and versus NF (## *p* < 0.01, ### *p* < 0.001).

As a comparison, we evaluated the combination of free spermidine with nanofenretinide and, also, in this case, we observed increased activity at the concentrations that triggered cytotoxicity in single administrations: 1500 µM and 2000 µM spermidine (Figure 7).

**Figure 8:**
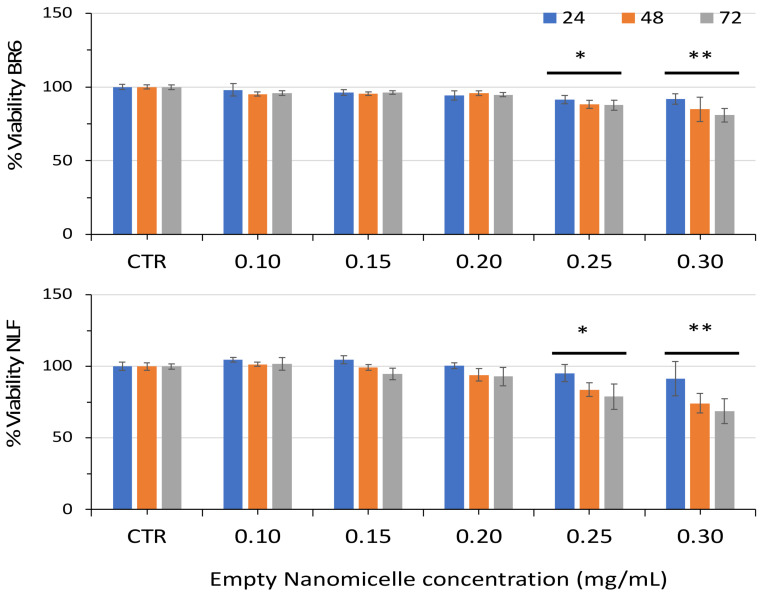
Relative viability of BR6 and NLF cells treated with Empty Nanomicelles at increasing concentrations for 24, 48, and 72 h assessed by MTT assay. Data are presented as percentage versus control cells (100%) (mean ± SD, *n* = 6) (* *p* < 0.05, ** *p* < 0.01).

To exclude any contribution of the nanomicelles to the improved cytotoxicity of nanospermidine, we evaluated the effect of empty nanomicelles on cell viability. No decrease in cell vitality was observed up to 0.2 mg/mL nanomicelles, corresponding to the maximum concentration used in this study. However, higher concentrations induced a slight but significant decrease in cell viability in both cell lines (Figure 8).

**Figure 9:**
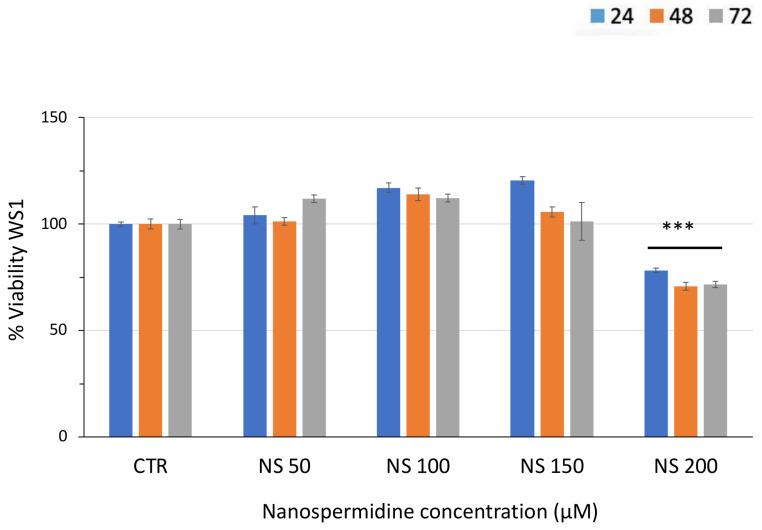
Relative viability of WS1 fibroblasts treated with increasing concentrations of Nanospermidine (NS) for 24, 48, and 72 h assessed by MTT assay. Data are presented as percentage versus control cells (100%) (mean ± SD, *n* = 6) (*** *p* < 0.001).

Finally, the effect of nanospermidine was evaluated in normal WS1 fibroblasts at the same concentrations used in the tumor cells (Figure 9). No decrease in viability was obtained up to 150 µM spermidine, corresponding to the cytotoxic concentration in tumor cells. At 200 µM, a 28.36% viability decrease was observed after 72 h.

### 3.3. Quantitative Phase Imaging

QPI evaluated cell morphology over time, providing, at the same time, measures of several parameters such as shape, dry mass, and sphericity, as well as cellular proliferation and displacement in response to treatment [28,29].

**Figure 10:**
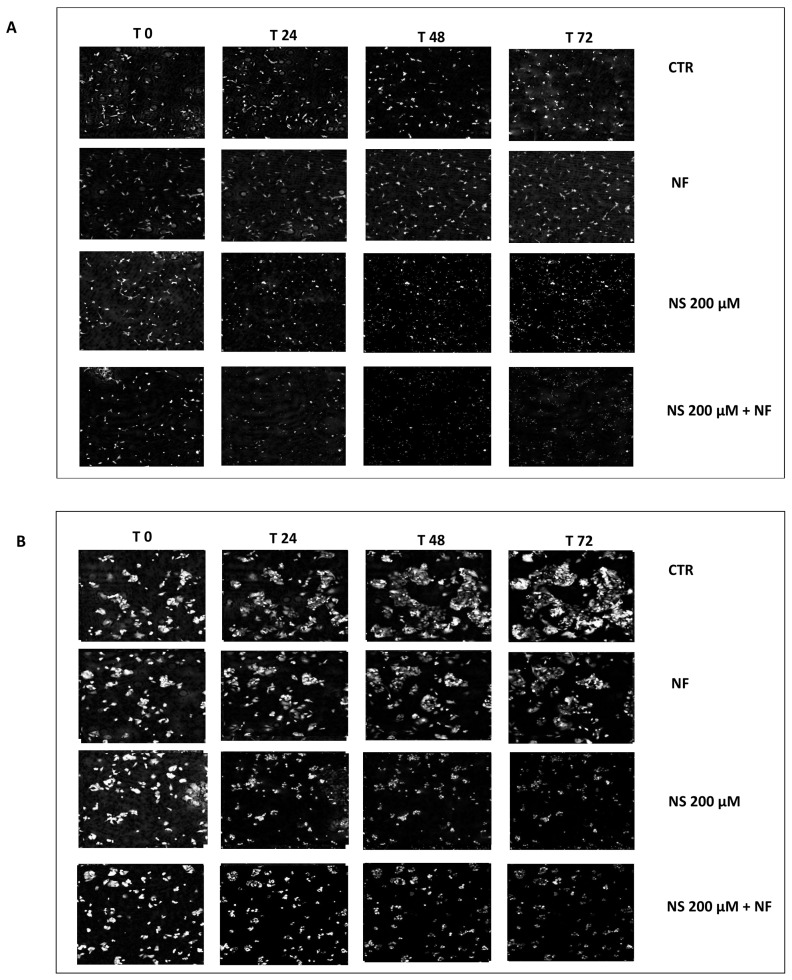
QPI. Representative image at 0, 24, 48, and 72 h of BR6 cells (**A**) and NLF cells (**B**) treated with Nanospermidine (NS, 200 μM spermidine) as a single agent and in combination with Nanofenretinide (NF, 10 µM fenretinide). As a comparison, the images of the untreated cells and the cells treated with NF are shown.

The images (Figure 10A,B) were obtained at up to 72 h of treatment with nanospermidine, and the combination with nanofenretinide confirmed the MTT results, indicating cytotoxic effects at 200 µM with a higher activity of the combination than single nanospermidine.

Cell doubling time, displacement, dry mass, and sphericity were analyzed over 72 h at the sub-cytotoxic concentrations 50 µM and 100 µM nanospermidine to exclude the interfering effect of cell death with detachment from the substrate.

**Figure 11:**
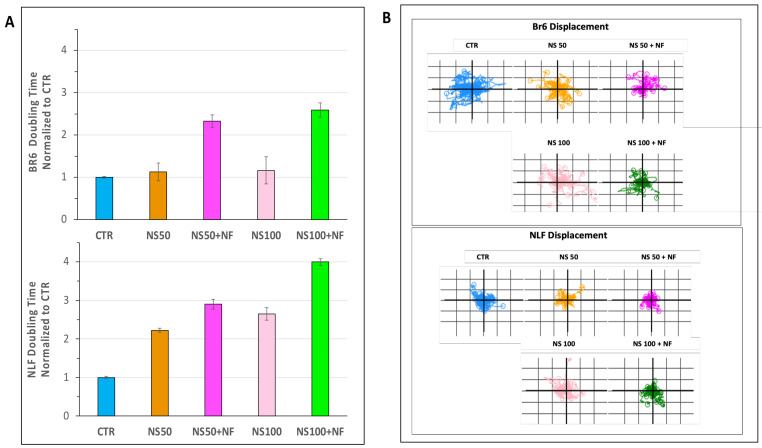
QPI data. (**A**) Histogram plot illustrating median cell doubling time of BR6 and NLF cells treated with nanospermidine (NS) and nanospermidine in combination with nanofenretinide (NS + NF) at the sub-cytotoxic concentrations of NS: 50 µM and 100 µM. (**B**) Displacement of BR6 and NLF cells treated with nanospermidine (NS) and nanospermidine in combination with nanofenretinide (NS + NF) at the sub-cytotoxic concentrations of NS: 50 µM and 100 µM.

The doubling time, which is a measure of cell growth, was increased by the combination in both cell lines, with a higher effect at 100 µM than 50 µM (Figure 11A), indicating a slowing down in cell proliferation.

Cell motility is obviously an important feature of tumor cells as it contributes to cancer invasion and metastasis [51,52]. Evaluation of cell displacement is now gaining increasing attention with improvements in time-lapse microscopy techniques [53].

In our study, we observed that cell displacement was decreased by nanospermidine, and the presence of fenretinide further limited cell displacement (Figure 11B).

In QPI, dry mass is measured from the phase delay considering that the refractive increment of biomolecules can be closely approximated by a constant [25].

A decrease in dry mass over time is indicative of a cytostatic or cytotoxic effect [54]. Sphericity quantifies the degree of cell roundness and is calculated as the ratio of the surface area of a sphere that has the same volume as the cell to the surface area of the cell. For adherent cells, such as those used in our study, sphericity provides a measure of the level of attachment of the cells to the substrate, since when the level of attachment increases the cells lose sphericity [55].

**Figure 12:**
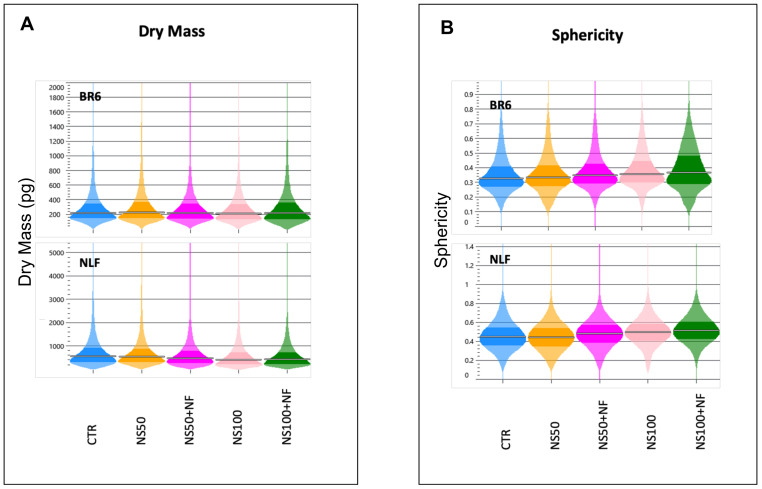
QPI data. (**A**) Dry mass at 72 h of BR6 and NLF cells treated with Nanospermidine (NS) and Nanospermidine in combination with Nanofenretinide (NS + NF) at the sub-cytotoxic concentrations of NS: 50 µM and 100 µM. (**B**) Sphericity at 72 h of BR6 and NLF cells treated with nanospermidine (NS) and nanospermidine in combination with nanofenretinide (NS + NF) at the sub-cytotoxic concentrations of NS: 50 µM and 100 µM. Analysis of cell doubling time, displacement, and dry mass was mediated on six wells for each treatment.

Treatment with the sub-cytotoxic concentrations of nanospermidine did not provide appreciable differences in dry mass (Figure 12A) and sphericity (Figure 12B) with respect to the controls. These results indicate that, despite the decrease in cell proliferation, the sub-cytotoxic concentrations of nanospermidine did not induce morphological variations.

Combination treatments, on the contrary, slightly decreased dry mass and increased sphericity in both cell lines, indicating a cytotoxic effect induced by fenretinide.

### 3.4. ROS Increase in Treated Cells

**Figure 13:**
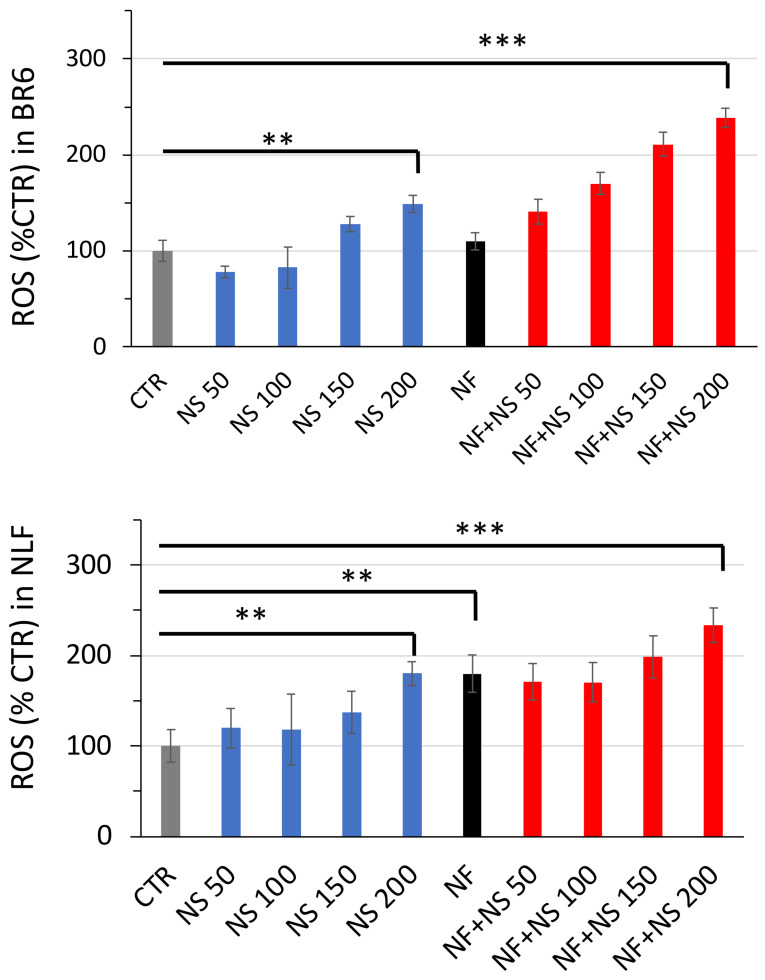
Effect of Nanospermidine (NS) and Nanospermidine in combination with Nanofenretinide (NS + NF) on ROS increase. ROS production was measured by H_2_DClFDA fluorescence in BR6 and NLF cells treated 4 h with Nanospermidine at concentrations corresponding to 50, 100, 150, and 200 µM spermidine or with Nanospermidine in combination with Nanofenretinide at 0.05 mg/mL corresponding to 10 µM fenretinide. Data are presented as percentage versus control cells (100%) (mean ± SD, *n* = 6) (** *p* < 0.01, *** *p* < 0.001).

Treatment with nanospermidine elicited ROS increases in both tumor cell lines. ROS increase was proportional to nanospermidine concentration (Figure 13). The highest ROS levels were obtained in NLF at 200 µM nanospermidine.

The presence of fenretinide further increased ROS at each nanospermidine concentration analyzed. At 200 µM nanospermidine, fenretinide provided comparable ROS levels in both cell lines.

### 3.5. Confocal Laser-Scanning Fluorescence Microscopy

**Figure 14:**
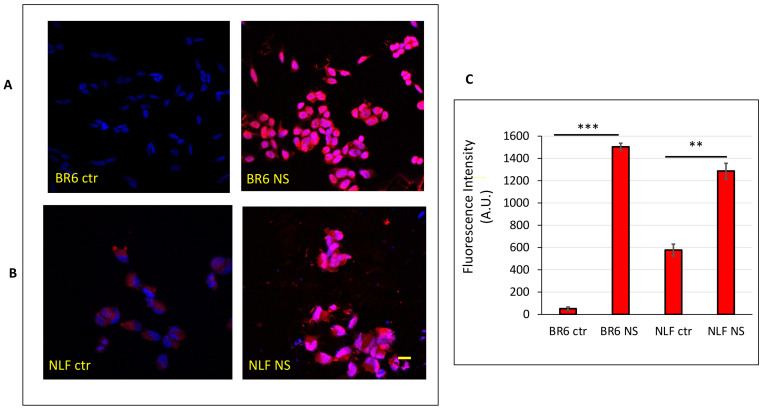
Confocal microscopy of BR6 (**A**) and NLF (**B**) after 15 min treatment with Nanospermidine (NS) stained with Nile Red and controls treated with Nile Red solution at the same concentration used with NS. Cell nuclei were evidenced by 1 μg/mL Hoechst 33348 staining. (**C**) Fluorescence intensities of the treated cells and controls. Photographs were taken at 40× magnification, bar = 20 µm. The images were analyzed by Image J Software and reported as fluorescence intensity per unit area. (mean ± SD, *n* = 3) (** *p* < 0.01, *** *p* < 0.001).

Cells treated with Red Nile stained nanospermidine were analyzed by confocal laser-scanning fluorescence microscopy. The images (Figure 14A) showed that nanospermidine can interact with the cells after short periods of time. The images reveal a uniform distribution of fluorescence in BR6 and a spotted-like dispersion in NLF. The quantitative analysis of fluorescence intensity (Figure 14B) showed that nanospermidine interaction was higher in BR6 than in NLF. WS1 did not provide fluorescent images after treatment with Red Nile stained nanospermidine for the same time period as the tumor cells.

## 4. Discussion

The increased polyamine metabolism and its correlation with cancer have prompted many efforts at developing drugs that could inhibit polyamine biosynthesis or block the active, carrier-mediated transport of polyamine into the cells as a therapeutic approach in cancer treatment.

More recently, attention has been paid to molecules that, on the contrary, upregulate polyamine catabolism in cells as a means to generate toxic compounds that can induce cancer cell death [7]. In fact, it has been demonstrated that the production of H_2_O_2_ through polyamine catabolism is implicated in the cytotoxic responses of several types of tumors to different polyamine analogs [8].

Among the molecules currently used to upregulate polyamine catabolism, polyamine analogs, such as N1,N11-diethylnorspermine (DENSpm) and N1-ethyl-N11-[(cyclopropyl)methyl]-4,8- diazaundecane (CPENSpm), have demonstrated activity in several types of tumors [9,11,12]. However, these molecules showed some drawbacks, such as poor specificity for cancer cells, induction of epithelial-mesenchymal transition-dedifferentiation in non-cancerous cells, and limited activity in clinical trials [11,12].

Therefore, a new approach to stimulate polyamine catabolism might rely on increasing intracellular polyamine concentrations above their physiological values to trigger the production of oxidation metabolites at levels exceeding cell tolerance. However, exogenous administration of free polyamines cannot raise their intracellular concentrations over physiological levels because intracellular penetration is strictly regulated by import-export mechanisms based on specific membrane carriers, such as SLC22A16 and SLC3A2, that import or export polyamines depending on the cell necessity [13]. In addition, the carriers’ activity is regulated by a feedback mechanism based on the immediate synthesis of antizyme, a regulatory protein that blocks the polyamine uptake in the presence of small increases in free cellular polyamine levels [14].

Other mechanisms of cell penetration, such as passive diffusion or endocytosis, are ineffective compared to the carrier-mediated active transport. Passive diffusion, in particular, is strongly hindered by the high intracellular polyamine concentrations in the mM range that sustain a negative outside-inside concentration gradient, being the physiological concentration of extracellular polyamines in the µM range. In addition, the extensive protonation of spermidine (pKa 10.9) at physiological pH reduces the concentration of the diffusible, unprotonated molecules to about 0.03% of the total extracellular concentration [14,15,16].

Furthermore, the extracellular concentration of polyamines is physiologically regulated to prevent accidental increases that could trigger an uncontrolled influx of polyamines into the cells driven by passive diffusion.

The control of extracellular polyamine concentration starts in the intestine where polyamines from the diet or produced by luminal bacteria are absorbed by a saturation regulated mechanism and are metabolized in the enterocytes before reaching the systemic circulation [56].

With the aim to overcome these limitations and obtain supra-physiological levels of spermidine in tumor cells, we prepared nanospermidine by encapsulation of spermidine in nanomicelles that we had previously demonstrated to be suitable carriers for fenretinide in tumor cells [21,22,23,24]. Next, we evaluated the effect of nanospermidine in two neuroblastoma cell lines: NLF (with MYCN amplification) and BR6 (without MYCN amplification, with TrKA expression).

A significant decrease in cell viability was obtained in both cell lines with nanospermidine concentrations of 0.15 mg/mL corresponding to 150 μM spermidine. With free spermidine, on the contrary, no cytotoxic effects were observed up to 1200 μM, and a slowing of cell proliferation in NLF and a complete inhibition in BR6 were obtained at 1500 μM.

The results were in agreement with the ability of the nanomicelles to transport the encapsulated spermidine within the cells, previously demonstrated for fenretinide encapsulated in the same nanomicelles [21,22] and supported by confocal laser-scanning fluorescence microscopy showing increased fluorescence in cells treated with Red Nile stained nanospermidine compared to controls treated with the free dye.

Cytotoxicity of empty nanomicelles was excluded by comparative experiments, indicating no cytotoxic effects at nanomicelle concentrations higher than those used in this study. Moreover, the cytotoxicity of nanospermidine was higher in tumor cells than in normal cells, such as WS1 fibroblasts, in accordance with the increased sensitivity of tumor cells to ROS increase compared to their normal counterparts. In fact, it has been demonstrated that cancer cells have ROS levels higher than normal cells, and their redox system is easily overwhelmed by additional ROS production that induces oxidative stress leading to cell death [57,58].

Based on this feature, many strategies are being developed to raise ROS levels and realize targeted therapies by induction of cell death in tumors without relevant toxicity to normal cells [59].

The tenfold higher cytotoxicity of nanospermidine than free spermidine suggested the suitability of the nanomicelles as polyamine transporters and revealed the efficiency of this mechanism to provide the cell with high amounts of spermidine owing to the favorable spermidine loading (12.92% *w*/*w*) in the nanomicelles. On the contrary, the much higher concentration of free spermidine required to trigger cytotoxicity correlates with the difficulty for the free molecule to enter the cells in amounts exceeding physiological values, evading cell transport regulation. These results denote the unsuitability of free spermidine as an antitumor agent because the high concentrations required at the tumor site would be hardly achieved even by the administration of large doses.

Moreover, the results obtained indicate that nanospermidine can be regarded as a new tool to increase intracellular polyamines levels and induce tumor cell death by using spermidine at biocompatible doses. In addition, the in-vivo administration of nanospermidine could exploit the mechanism of nanomedicines accumulation at the tumor site by extravasation through the discontinuities of the capillaries generated by angiogenesis, providing on-target activity. The ability of nanospermidine to increase its size at high dilutions could represent an additional favorable feature for targeted localization. Indeed, in the presence of high dilutions, such as those provided by the tumor matrix, the size expansion of the extravasated nanoparticles could prevent their retro-diffusion to the venous circulation that, at present, is recognized as a drawback in passive drug targeting of nanomedicines [60].

We found a correlation between nanospermidine cytotoxicity and increased intracellular ROS levels. This prompted us to evaluate the combination of nanospermidine with nanofenretinide, as we had previously demonstrated that nanofenretinide was endowed with high antitumor activity towards these same cell lines [21,22] due to ROS increase.

Nanofenretinide improved the effect of nanospermidine at both cytotoxic and sub-cytotoxic nanospermidine concentrations. It increased cell death at the cytotoxic concentrations while, at the sub-cytotoxic concentrations, it decreased cell proliferation, as indicated by prolonged duplication times and decreased cell motility.

Furthermore, cotreatment with nanofenretinide at the sub-cytotoxic nanospermidine concentrations slightly increased sphericity and decreased biomass. These slight but important modifications were disclosed by time-lapse QPI techniques that measured several parameters at the same time and provided a multifaced image of the cells. They indicated that, while treatment with nanospermidine at sub-cytotoxic concentrations did not influence cell morphology, cotreatment with nanofenretinide may induce apoptosis, even at such low nanospermidine concentrations, as suggested by the reduced biomass and the increased sphericity indicative of decreased cell attachment. This information could not have been obtained by other techniques such as MTT assay.

## 5. Conclusions

Nanospermidine, obtained by spermidine encapsulation in nanomicelles, can represent a new tool in antitumor therapy being endowed with both the pharmacokinetics characteristics of nanomedicines and the activity of polyamines catabolism modulators.

As a nanocarrier, nanospermidine can undergo accumulation at the tumor site by extravasation through the discontinuities of the tumor capillaries, thus allowing spermidine localization at the pathological site.

At the cellular level, nanospermidine can provide spermidine penetration into the cells in amounts exceeding physiological values, thus increasing ROS production over limiting values for cell tolerance and triggering cell death.

Administration of nanospermidine in combination with nanofenretinide further increases the antitumor activity of nanospermidine.

Hence, nanospermidine may represent a new approach in tumor treatment because it provides a high intracellular increase of the polyamine and tumor cell death by administration of micromolar, physiological doses of spermidine that make the treatment tolerable and biocompatible.

Finally, the proven ability of spermidine to activate macrophages and memory B cell responses makes nanospermidine worth further evaluation to assess if the antitumor effect demonstrated in this study can be further improved by inhibition of tumor immunosuppression in in vivo settings.

## Figures and Tables

**Table 1:** Physicochemical Characteristics of Nanospermidine (NS), Nanofenretinide (NF), and empty nanomicelles (No) in PBS at 50 mg/mL.

Nanomicelle Type	% Loading (*w*:*w*)	Mean Size (nm)	Polydispersity	Zeta Potential (mV)
**NS**	Spermidine 12.92 ± 2.37	148.4 ± 3.4	0.270 ± 0.013	−17.4 ± 0.57
**NF**	Fenretinide 7.82 ± 1.05	154.1 ± 10.3	0.258 ± 0.011	−27.2 ± 1.53
**No**	_	124.7 ± 3.5	0.183 ± 0.024	−21.7 ± 2.18

## Data Availability

The data presented in this study are available upon request from the corresponding author, I.O.
